# Interleukin 4 improved adipose-derived stem cells engraftment via interacting with fibro/adipogenic progenitors in dystrophic mice

**DOI:** 10.1007/s00018-023-05020-2

**Published:** 2023-11-27

**Authors:** Huan Li, Jinfu Lin, Liang Wang, Ruojie He, Jing Li, Menglong Chen, Weixi Zhang, Cheng Zhang

**Affiliations:** 1 Department of Neurology, The First Affiliated Hospital, Guangdong Provincial Key Laboratory of Diagnosis and Treatment of Major Neurological Diseases, National Key Clinical Department and Key Discipline of Neurology, Sun Yat-Sen University, No. 58 Zhongshan Road 2, Guangzhou, 510080 China; 2 Department of Neurology, The First Affiliated Hospital of Shantou University Medical College, Shantou, 515000 China; 3 Department of Neurology, The First Affiliated Hospital, Jinan University, Guangzhou, 510080 China

**Keywords:** Dystrophinopathy, Adipose-derived stem cells, IL4, Fibro-/adipogenic progenitors, Muscle fibrosis

## Abstract

Adipose-derived stem cells (ADSC) therapy shows promise as an effective treatment for dystrophinopathy. Fibro-/adipogenic progenitors (FAPs) play an essential role in the myogenesis of muscle satellite cells and contribute to muscle fibrosis and adipocyte infiltration. The interleukin 4 (IL-4) pathway acts as a switch that regulates the functions of FAPs. The interaction between FAPs and engrafted cells remains unclear. In this study, we used a co-culture system to investigate possible crosstalk between the FAPs of dystrophic mice and ADSC overexpressing IL4 (IL4-ADSC) and control ADSC. Systemic transplantation of IL4-ADSC and control ADSC in dystrophic mice was conducted for 16 weeks, after which motor function and molecular improvements were evaluated. Overexpression of IL4 in ADSC significantly promoted myogenesis in vitro, increasing the expression of Pax7, Myogenin, and MyHC. Co-culture indicated that although myoblasts derived from control ADSC promoted adipogenic and fibrogenic differentiation of FAPs, FAPs did not significantly affect myogenesis of ADSC-derived myoblasts. However, overexpression of IL4 in ADSC inhibited their myotube-dependent promotion of FAPs differentiation on the one hand and promoted FAPs to enhance myogenesis on the other. Dystrophic mice administered with IL4-ADSC-derived myoblasts displayed significantly better motor ability, more engrafted cells showing dystrophin expression, and less muscle fibrosis, intramuscular adipocytes, and macrophage infiltration than mice administered control-ADSC-derived myoblasts. In conclusion, IL4 activation enhanced the therapeutic potential of ADSC transplantation in dystrophic mice, possibly by improving the myogenesis of IL4-ADSC and altering the crosstalk between engrafted stem cells and resident FAPs.

## Introduction

Duchenne muscular dystrophy (DMD), an X-linked, degenerative muscular disorder characterized by progressive muscle weakness, affects 1 in 3500 newborn males. Mutations in the *DMD* gene leading to defective dystrophin cause DMD. Progressive pathological changes in muscles due to fibrosis and fatty infiltration are hallmarks of advanced DMD [1, 2]. Currently, there is no curative therapy for DMD. Inhibition of muscle fibrosis and fatty infiltration are potential treatments for DMD. Skeletal muscle fibrosis in DMD is characterized by the accumulation of type I and III collagen [3]. Heterogeneous cell types, including fibro-/adipogenic progenitors (FAPs) and cells of endothelial or hematopoietic origin, reportedly cause fibrosis via fibrogenic conversion [4–6]. Recent studies have proposed that FAPs are the primary source of fibrillar collagen matrix synthesis in dystrophic muscles [6, 7]. These non-myogenic cells, which are positive for cell-surface platelet-derived growth factor receptor-α (PDGFR-α) and stem cell antigen 1 (Sca1), remain quiescent in normal muscles but proliferate efficiently in response to acute damage and differentiate into collagen via cycles of degeneration or regeneration, causing the intramuscular fibrosis seen in muscular dystrophy [7, 8]. Therapies that target altered FAPs activities have potently inhibited fibrosis in muscular dystrophy, indicating that FAPs are potential targets for preventing fibrosis in DMD [9, 10]. FAPs were also found to be the primary cellular source of intramuscular adipocytes [11, 12], and therapies aimed at altering the adipogenesis of FAPs could efficiently limit fatty degeneration in dystrophic muscles [13].

FAPs also play a positive role in skeletal muscle regeneration and homeostatic maintenance. FAPs isolated from regenerating muscles promote the proliferation and myogenic differentiation of satellite cells (SCs) in co-culture [8, 11]. Regenerative deficits in muscle, muscle atrophy, and loss of expansion of SCs were observed following FAPs depletion [14]. Overall, the function and fate of FAPs act as a two-edged sword in muscle regeneration. Mediating a balance between the ability of FAPs to induce myogenesis and their ability to facilitate intramuscular fibrosis and adipocyte formation is crucial. Studies have found that IL4 signaling serves as a key switch in regulating the function and fate of FAPs [15, 16]. Upon injury, activation of IL4 signaling induces FAPs to initiate promoting myogenesis while simultaneously inhibiting the differentiation of FAPs into adipocytes [15, 16].

Furthermore, IL4 signaling plays a role in myogenesis. IL4 signaling influenced myogenesis by stimulating the fusion of SC-derived-myoblasts and improving the migration of myogenic precursor cells [17, 18]. Moreover, IL4 stimulated the myogenic differentiation of C2C12-derived-myoblasts by inducing myoblast fusion and rescuing myogenesis, which improved the muscle mass and performance of colon carcinoma-bearing mice [19]. However, whether IL4 signaling influences myogenesis in dystrophic muscle and stem cell differentiation remains unclear.

Stem cell therapy is considered a candidate therapy for DMD. Adipose-derived stem cells (ADSC) can engraft into dystrophic muscle, generating dystrophin-expressing myofibers and successfully replenishing the pool of SCs [20]. The mechanism underlying ADSC-based therapy in mdx mice remains unclear. ADSC show great potential for tissue repair and regeneration, possibly by releasing specific factors, such as VEGF, HGF, IGF, and IL-10, which are associated with regenerative and anti-inflammatory effects [21]. IL4 reportedly promoted the proliferation of ADSC and their fusion with myoblasts in vitro [22, 23]. However, reports describing the possible relationship between IL4 and ADSC following transplantation in dystrophic muscle are scant. Besides, muscle homeostasis is determined by reciprocal functional interactions between cell types, including myofibers, SCs, FAPs, and specific infiltrated immune cells, and recent evidence has demonstrated that complicated crosstalk occurs between them [24]. However, reports regarding studies that investigated possible crosstalk between FAPs and engrafted stem cells are currently unavailable.

This study aimed to investigate possible interactions between resident FAPs and ADSC-derived myoblasts as well as the possible role played by the IL4 pathway in these interactions via co-culture in vitro. An in vivo transplantation study was conducted to verify whether targeting IL4 signaling would improve ADSC engraftment in dystrophic mice. To the best of our knowledge, this is the first study to describe possible interactions between muscle resident FAPs and engrafted cells to illustrate the therapeutic potential of IL4 in combination with cell therapy for DMD.

## Methods

### Animals

Mdx mice (C57BL/10ScSn-DMDmdx/J) were purchased from the Jackson Laboratory (Bar Harbor, ME, USA). Wild-type C57BL/6 mice were purchased from the Guangdong Medical Laboratory Animal Center (Guangzhou, China). The mice were raised in a specific pathogen-free animal facility according to standard procedures. Animal protocols were approved by the Animal Care and Experimentation Committee of Sun Yat-Sen University (SYSU-IACUC-2018-000199).

### Cell preparation and fluorescence-activated cell sorting (FACS) isolation

The previously described methodology was used for isolating mouse ADSC [20]. We used C57BL/6 mice (4 weeks old) to isolate ADSC. Briefly, adipose tissue from the inguinal fat deposits of mice was carefully separated and digested using 0.1% collagenase I (Invitrogen, Carlsbad, CA, USA). The acquired cell suspension was cultured in growth media containing Dulbecco’s modified Eagle’s medium (DMEM)/F-12 (Gibco, Grand Island, NY, USA) and 10% fetal bovine serum (FBS) (Gibco).

Isolation of FAPs was performed according to the protocol of a previous study [15]. The bilateral hind limb muscles of 8-week-old mdx mice were separated and digested in media containing DMEM, 800 U/ml collagenase II (Gibco), and 0.3 U/ml dispase (Gibco). Single-cell samples were collected and incubated with the following primary antibodies (eBioscience, Grand Island, NY, USA) for 30 min at 4 °C: CD45, CD31, CD11b, Sca1, and ITGA7. Next, 7-Aminoactinomycin D (7-AAD, eBioscience) was used to identify live/dead cells. Sorting was performed on MoFlo Astrios EQs (Beckman Coulter Inc., Fullerton, CA, USA). FAPs acquired via FACS were seeded on cell culture clusters in growth media containing DMEM/F-12 medium, 20% FBS, 2.5 ng/ml bFGF (Invitrogen) on plates previously coated with Matrigel (Corning).

### Adipogenic, osteogenic, and myogenic differentiation of ADSC

Adipogenic and osteogenic differentiation of ADSC has been described previously [25]. Myogenic differentiation was performed as previously described [20]. Briefly, we cultured ADSC in myogenic induction medium [DMEM containing 10% FBS, 0.5 μM BIO (Santa Cruz, Dallas, TX, USA), 20 μM forskolin (Santa Cruz), and 10 ng/ml fibroblast growth factor-basic (bFGF) (PeproTech, Rocky Hill, NJ, USA)] for 7 days. Then, we replaced it with a myogenic maintenance medium (DMEM supplemented with 2% horse serum) for further induction.

### Construction of IL4 expressing plasmids and infection of lentiviral vectors

Vehicle lentivirus (pLent-EF1a- Green fluorescent protein (GFP)-P2A-Puro) and IL4-overexpressing lentivirus (pLent-EF1a-GFP-P2A-Puro-CMV-IL4) were purchased from OBiO Technology (Shanghai, China). We transduced ADSC with lentiviruses for 12 h at a multiplicity of infection (MOI) of 60 and 6 μg/mL polybrene (OBiO Technology) was used for transduction. After 72 h of transduction, we selected the transducted ADSC with 2 μg/mL puromycin for 48 h. Cells were classified into two treatment groups: ADSC transduced with vehicle lentivirus (VEH-ADSC) and ADSC transduced with IL4-overexpressing lentivirus (IL4-ADSC).

### Co-culture of ADSC-derived myoblasts and FAPs

A Transwell (Corning, Midland, MI, USA) chamber with a 0.4 µm pore polyester membrane insert was used for co-culture. IL4-ADSC and VEH-ADSC inducing myogenic differentiation for 14 days were used in the co-culture study and they were digested into single cells by 0.25% Trypsin–EDTA, as previously described [20]. A similar number of ADSC-derived myoblasts and FAPs were seeded in the transwell chambers for co-culturing.

To analyze the effects of ADSC-derived myoblasts on the differentiation ability of FAPs, ADSC-derived myoblasts and FAPs were co-cultured in a myogenic maintenance medium and an adipogenic induction medium consisting of DMEM, 10%FBS and 1 μg/ml insulin for 5 days. FAPs co-cultured with ADSC-derived myoblasts, including FAPs (Co-cult with IL4-ADSC) and FAPs (Co-cult with VEH-ADSC) were used for analyzing, using mono-cultured FAPs under similar conditions as control. Besides, we added IL4 (2.4 µg/ml) to the medium consisting of DMEM, 10% FBS, and 1 µg /ml insulin for 5 days, and the group (FAPs + IL4) was then compared with mono-cultured FAPs to confirm whether IL4 influences the differentiation ability of FAPs.

To study the effect of FAPs on the myogenesis of ADSC-derived myoblasts, ADSC-derived myoblasts and FAPs were co-cultured in a myogenic maintenance medium and a medium consisting of DMEM, 10% FBS for 14 days. Following 14 days of co-culturing, the IL4-ADSC and VEH-ADSC-derived myotubes (named IL4-ADSC (Co-cult with FAPs) and VEH-ADSC (Co-cult with FAPs)) and FAPs (named FAPs (IL4-ADSC Co-cult) and FAPs (VEH-ADSC Co-cult)) in the co-culture system were used for further analysis. Transwell chambers were coated with 0.1% gelatin. The medium was changed every 2 days.

### Enzyme-linked immunosorbent assay (ELISA)

IL4 levels in the cellular supernatant were analyzed via ELISA. Generally, we digested ADSC into single cells. Then, 1*10^6^ cells were planted in a 35 mm cell culture plate (Corning), and a 2 ml culture medium or myogenic induction medium was added to each plate. The cellular supernatant of undifferentiated ADSC was collected after culturing for 24 h, and the cellular supernatants of differentiated ADSC were collected on days 14 and 28 after myogenic differentiation. All the cellular supernatants were analyzed using a Mouse IL4 Quantikine ELISA Kit (R&D Systems, Minneapolis, MN, USA) according to the manufacturer's instructions.

### Cell counting kit 8 (CCK8) assays

A cell Counting Kit 8 (Abcam) was used to access the proliferation of IL4-ADSC and VEH-ADSC. Generally, 10,000 cells per well were plated in a culture microplate. Following 24, 48, and 72 h of culturing, 10 µl of CCK8 solution was added to each well (96-well plate) under dark conditions and incubated for 4 h at 37 °C. Absorbance at OD = 450 nm was measured.

### Transplantation of ADSC

Eight-week-old male mdx mice were used in this study. Myogenic differentiation was induced in VEH-ADSC and IL4-ADSC for 14 days, following which these cells were digested into single cells in PBS for transplantation purposes. Next, each mdx mouse was systemically administered 2 × 10^6^ cells in 200 μl PBS via injection into the tail vein to obtain mdx + VEH-ADSC and mdx + IL4-ADSC mice, respectively. In contrast, control mdx mice were injected with a similar volume of PBS (mdx + PBS). By contrast, wild-type C57/BL6 mice of the same age and gender were used as healthy controls. Following transplantation for 16 weeks, mice in these four groups were assessed for motor ability and then sacrificed to obtain tibialis anterior (TA) muscles, which were stored until needed for further analyses. Each group consisted of five mice (*n *= 5).

### Protein extraction and Western blotting

Whole-cell lysates were extracted using RIPA buffer (Thermo Scientific, Waltham, MA, USA) supplemented with a protease and phosphatase inhibitor Cocktail Kit (Thermo Scientific). Protein concentration was assessed using a BCA protein assay kit (Thermo Scientific). Sodium dodecyl sulfate-polyacrylamide gel electrophoresis (8% or 10%) was used to separate soluble proteins. Next, the proteins were transferred to polyvinylidene difluoride membranes (Millipore, Billerica, MA, USA), blocked, and incubated at 4 °C overnight with the primary antibodies: Myod1 (Santa Cruz), Myf5 (Abcam, Cambridge, MA, USA), Pax7 (Abcam), Myogenin (Abcam), MyHC (DSHB, Iowa City, IA, USA), STAT6 (CST, Danvers, MA, USA), pSTAT6 (CST), AKT (CST), pAKT (CST), ERK1/2 (CST), pERK1/2 (CST), β-Tubulin (CST), GAPDH (CST), αSMA (Sigma, St. Louis, MO, USA), Collagen III (Abcam), Collagen I (Abcam), Perilipin-1 (CST), PPARγ (CST), GFP (CST), INOS(Abcam), and CD206 (Abcam) followed by incubation with secondary antibodies for one hour at room temperature. Proteins were visualized using an enhanced chemiluminescence detection system (Thermo Fisher Scientific). Quantitative protein expression analysis was performed using Image J software (National Institutes of Health, Bethesda, MD, USA).

### Immunocytofluorescence and Immunohistofluorescence

Cultured cells were washed with PBS, fixed, permeabilized with 0.3% Triton X-100 (Sigma), blocked with 1% bovine serum albumin (BSA, Sigma), and incubated overnight at 4 °C with the following primary antibodies: Pax7 (Abcam), Myod1 (Abcam), Myf5 (Abcam), Desmin (Abcam), Dystrophin (Abcam), Myogenin (Abcam), MyHC (DSHB), IL4Rα (Abcam), and PDGFRα (CST), this was followed by incubation with secondary antibodies for one hour at room temperature. The fusion index was determined as the ratio between the number of nuclei (at least two) incorporated into myotubes determined by immunodetection of MyHC and the total number. Tissue samples were sliced into serial 8-μm frozen sections. Tissue sections were fixed with cold acetone, permeabilized, and blocked as previously described, and then incubated overnight with primary antibodies against Dystrophin (Abcam), GFP (Abcam), CD206(Abcam), and INOS(Abcam) at 4 °C, followed by incubation with secondary antibodies for one hour at room temperature. Fluoroshield™ with DAPI (Sigma) was used to stain the nuclei. Immunoreactivity was visualized and imaged using a NIKANG fluorescence microscope (Olympus, Tokyo, Japan).

### Quantitative polymerase chain reaction (qPCR)

Total RNA was extracted from cells using RNAiso (Takara Bio Inc., Otsu, Japan) following which cDNA was prepared using PrimeScript RT Master Mix (Takara Bio Inc.). Next, qPCR was performed using Tli RNaseH Plus SYBR Premix Ex Taq (Takara Bio Inc.) and the following forward and reverse primers: IL4, 5′-AAACGTCCTCACAGCAACGA-3′ and 5′- GCATCGAAAAGCCCGAAAGA-3′; IL-6, 5′-CCTACCCCAATTTCCAATGCTC-3′ and 5′-GGTCTTGGTCCTTAGCCACTC-3′; IGF-1, 5′-TGGCGCTCTGCTTGCTCACCTT-3′ and 5′-TAAAAGCCCCTCGGTCCACACACG-3′; Wnt1, 5′- CCCCACCTCTTCGGCAAGAT -3′ and 5′-ATGGAGCCTTCGGAGCAGGA-3′; Wnt3a, 5′-AAGTGGGGCGGCTGTAGTGA-3′ and 5′-TTCATGGCAGAGCGGGCATC-3′; Wnt5a, 5′-CCACTTGTATCAGGACCAC-3′ and 5′-TGTGCTGCAGTTCCATCTC-3′; and GAPDH, 5′-GCACCGTCAAGGCTGAGAAC-3′ and 5′- TGGTGAAGACGCCAGTGGA-3′. GAPDH was used as an internal control.

### Morphological studies

According to the manufacturer’s instructions, collagen deposition was detected via Masson’s trichrome staining (Solarbio, Beijing, China). Images were analyzed using Image J to quantify the fibrotic area-to-total area ratio. An average of five random fields of each section and three random sections of each mouse were used for the analysis. An Oil Red O stain kit (for cultured cells) (Solarbio) was used according to the manufacturer’s instructions. Images were analyzed using Image J to quantify the Oil Red O staining area. Five random detection fields were used, and three independent experiments were conducted.

### Motor ability assessment

Two distinctive hanging tests were used to assess the motor abilities of the mice [26]. For the hanging test involving two forelimbs, a metal cloth hanger was tightly attached to a shelter at 37 cm above ground height. The mouse was compelled to grasp the wire with only its two forelimbs, following which the mouse was released, and the timer started. For the hanging tests involving all four limbs, the lid of a big cage meant for rats was set tightly to a shelter at a height of 25 cm above ground. Each mouse was placed on the grid with all four paws grasping it, following which inverted the grid so that the mouse was hanging and directly starting the timer. The time spent hanging was recorded as the final time taken for all hanging limbs to drop. The hanging limit was fixed at 600 s. These two hanging tests were repeated thrice for each mouse, and the longest hanging time was used for the final analysis.

### Statistical analysis

Data were analyzed using SPSS (v.20.0; IBM Corp., Armonk, NY, USA) and GraphPad (v.8.0; GraphPad. Software, La Jolla, CA, USA). Normally distributed data are presented as mean ± standard deviation. The Student’s *t*-test and analysis of variance (ANOVA) were used to compare two groups and three or more groups, respectively. The Bonferroni test was used for comparisons between multiple groups.

## Results

### The characteristics of ADSC and overexpression of IL4 in ADSC

**Fig. 1:**
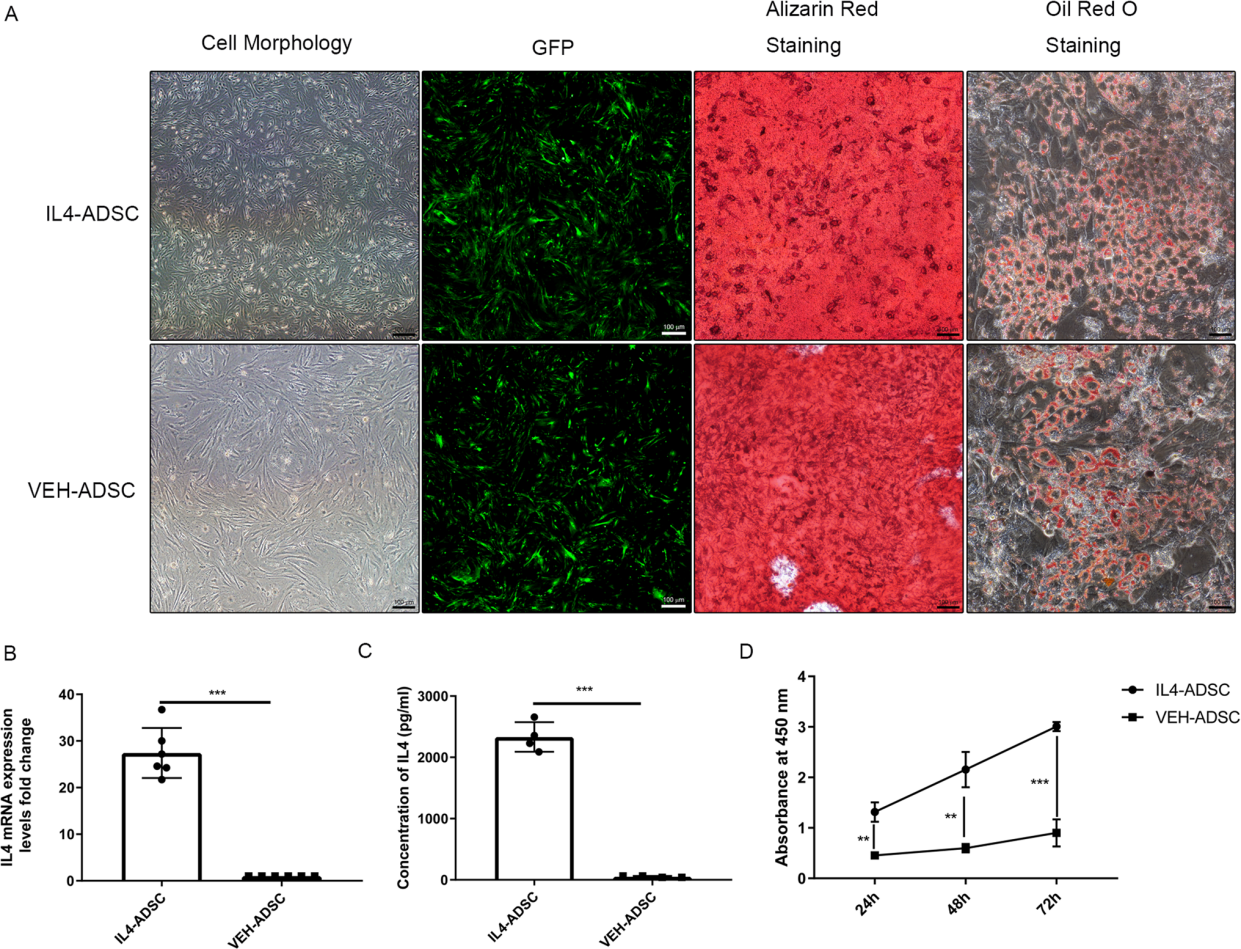
Establishment of IL4-overexpressing ADSC. **A** The cell morphology and GFP (green) expression of IL4-ADSC and VEH-ADSC. Osteogenic differentiation and adipogenic differentiation of IL4-ADSC and VEH-ADSC were confirmed by Alizarin Red and Oil Red O staining, respectively; scale bar, 100 μm. **B** IL4 mRNA levels in IL4-ADSC relative to VEH-ADSC. **C** The concentration of IL4 in cell supernatant of IL4-ADSC and VEH-ADSC, measured by the ELISA test. **D** Proliferation of IL4-ADSC and IL4-ADSC, measured by CCK8 assays; *P < 0.05, **P < 0.01, ***P < 0.001

The ADSC displayed a spindle-like morphology (Fig. 1A). GFP expression peaked following transduction with lentivirus for 72 h (Fig. 1A). Overexpression of IL4 did not change the adipogenic and osteogenic differentiation abilities of ADSC (Fig. 1A). We confirmed that IL4 mRNA expression in IL4-ADSC was significantly higher (28.26 ± 3.31-fold) than that of VEH-ADSC (Fig. 1B). ELISA indicated that the concentration of IL4 in the cellular supernatants of 1*10^6^ IL4-ADSC and VEH-ADSC after 24 h was 2332.00 ± 120.60 pg/ml, 52.45 ± 6.18 pg/ml, respectively (Fig. 1C). Thus, ELISA results and mRNA expression indicated that ADSC overexpressing IL4 had been successfully established. The CCK8 assays indicated that lentiviral transduction had promoted IL4 overexpression in ADSC (Fig. 1D).

### Overexpression of IL4 improves the myogenic differentiation ability of ADSC

**Fig. 2:**
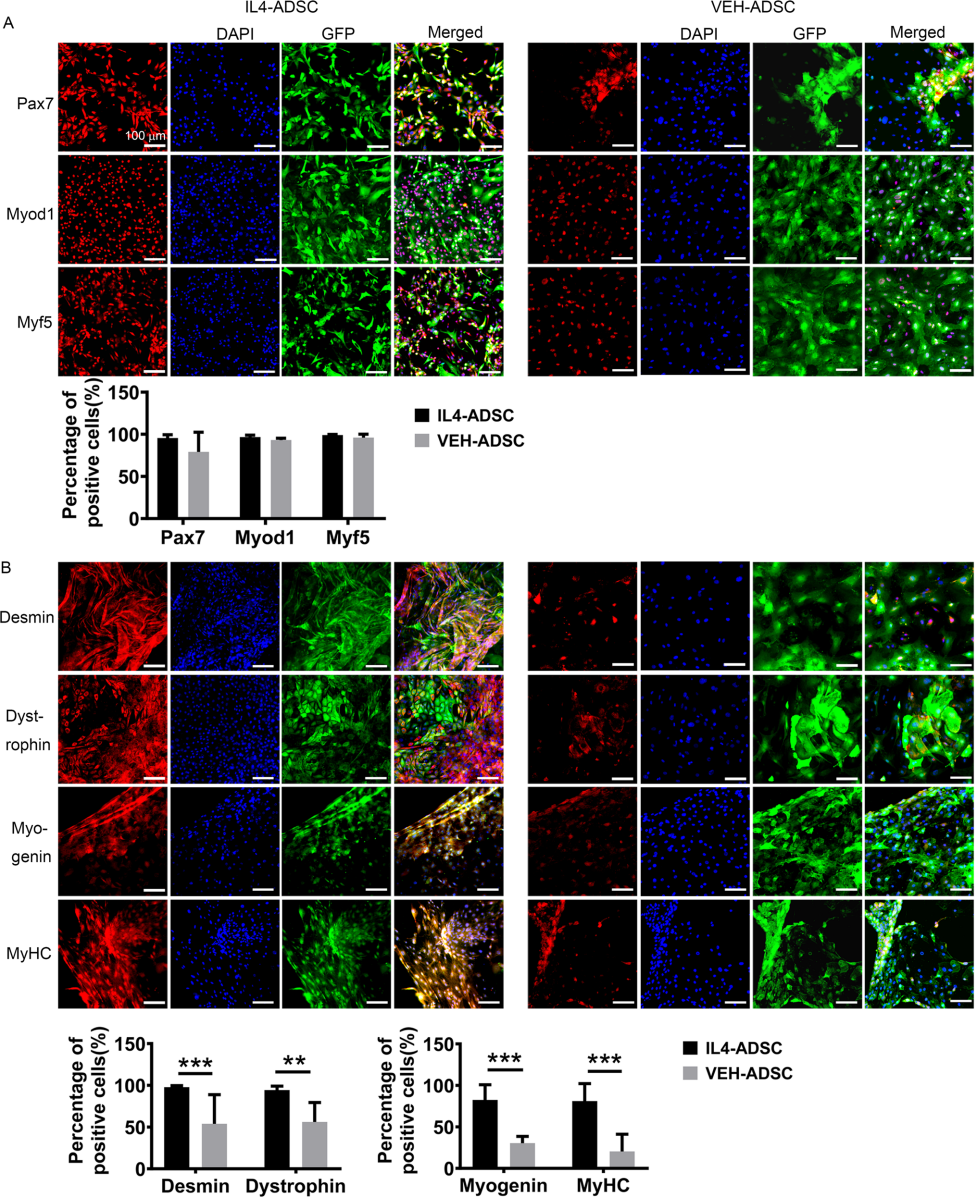
Immuno-cyto-fluorescence of myogenic marker expression at different time points following myogenic differentiation of IL4-ADSC and VEH-ADSC. **A** Expression of Pax7 (red), Myod1 (red), and Myf5 (red) in IL4-ADSC and VEH-ADSC following myogenic differentiation for 14 d. **B** Expression of Dystrophin (red), Desmin (red), Myogenin (red), and MyHC (red) in IL4-ADSC and VEH-ADSC following myogenic differentiation for 28 and 36 d; scale bar, 100 μm; *P < 0.05, **P < 0.01, ***P < 0.001

**Fig. 3:**
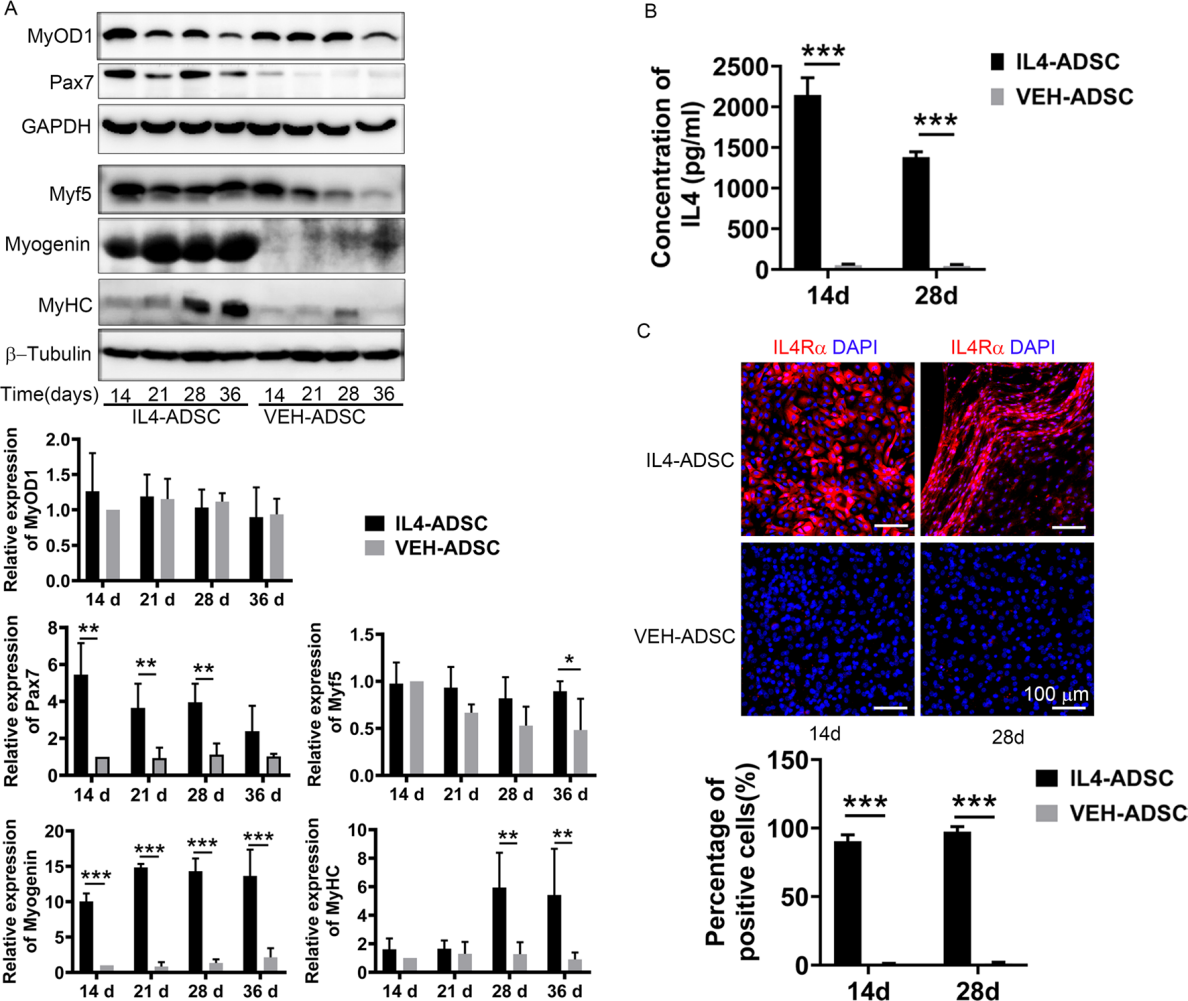
IL4-ADSC showed better myogenic differentiation capacity and increased IL4 pathway activity than VEH-ADSC. **A** Western blotting was performed to measure the expression of myogenic markers, Myod1, Pax7, Myf5, Myogenin, and MyHC in IL4-ADSC and VEH-ADSC at different time points (14, 21, 28, 36 d) of myogenic differentiation. Analyses of protein levels were related to β-tubulin (*n* = 4 each); **B** The concentration of IL4 in cell supernatants of IL4-ADSC and VEH-ADSC after myogenic differentiation for 14 and 28 days, measured by the ELISA test; **C** Immuno-cyto-fluorescence of IL4Rα expression in IL4-ADSC and VEH-ADSC after myogenic differentiation for 14 and 28 days; scale bar, 100 µm; *P < 0.05, **P < 0.01, ***P < 0.001

Pax7, Myod1, and Myf5 acted as early myogenic markers and immune-cyto-fluorescence revealed that they were expressed in both groups on day 14 following myogenic differentiation (Fig. 2A). The detection of terminal myogenic markers, including Dystrophin, Desmin, Myogenin, and MyHC, confirmed that both groups could be induced to undergo terminal myogenic differentiation (Fig. 2B). Immuno-cyto-fluorescence indicated that the expression levels of Dystrophin, Desmin, MyHC, and Myogenin in IL4-ADSC were higher than those in VEH-ADSC (Fig. 2B). The expression levels of Pax7, Myod1, Myf5, Myogenin, and MyHC at different time points were also analyzed via western blotting (Fig. 3A). Differences between the expression levels of Myod1 in IL4-ADSC and VEH-ADSC at different time points were not statistically significant (Fig. 3A). During days 14–28, the expression levels of Pax7 in IL4-ADSC were statistically higher than those in VEH-ADSC (Fig. 3A). Although the differences between the expression levels of Myf5 in IL4-ADSC and VEH-ADSC during days 14–28 were not statistically significant, the Myf5 level in IL4-ADSC on day 36 was higher compared to that in VEH-ADSC (Fig. 3A). Western blotting also indicated improved terminal myogenic differentiation in IL4-ADSC compared to VEH-ADSC. The expression levels of Myogenin in IL4-ADSC were significantly higher than those in VEH-ADSC at every time point (Fig. 3A). MyHC observed in IL4-ADSC on day 28 was increased even more by day 36; however, MyHC was not detected in VEH-ADSC at any time point by western blotting (Fig. 3A).

We also analyzed the expression of IL4 and IL4Rα in differentiated myogenic cells. ELISA indicated that the higher concentration of IL4 in the cellular supernatants of IL4-ADSC compared to that in VEH-ADSC after myogenic differentiation for 14 days (2146.13 ± 211.09 pg/ml vs. 52.45 ± 12.75 pg/ml) and 28 days (1382.38 ± 64.38 pg/ml vs. 43.93 ± 17.71 pg/ml) (Fig. 3B). Besides, the expression levels of IL4Rα were also increased in IL4-ADSC compared to those in VEH-ADSC after myogenic differentiation for 14 days and 28 days (Fig. 3C), illustrating the increased activity of IL4 pathway in IL4-ADSC during myogenic differentiation.

### IL4-ADSC-derived myoblasts inhibit adipogenic and fibrogenic differentiation of FAPs under a co-culture system

**Fig. 4:**
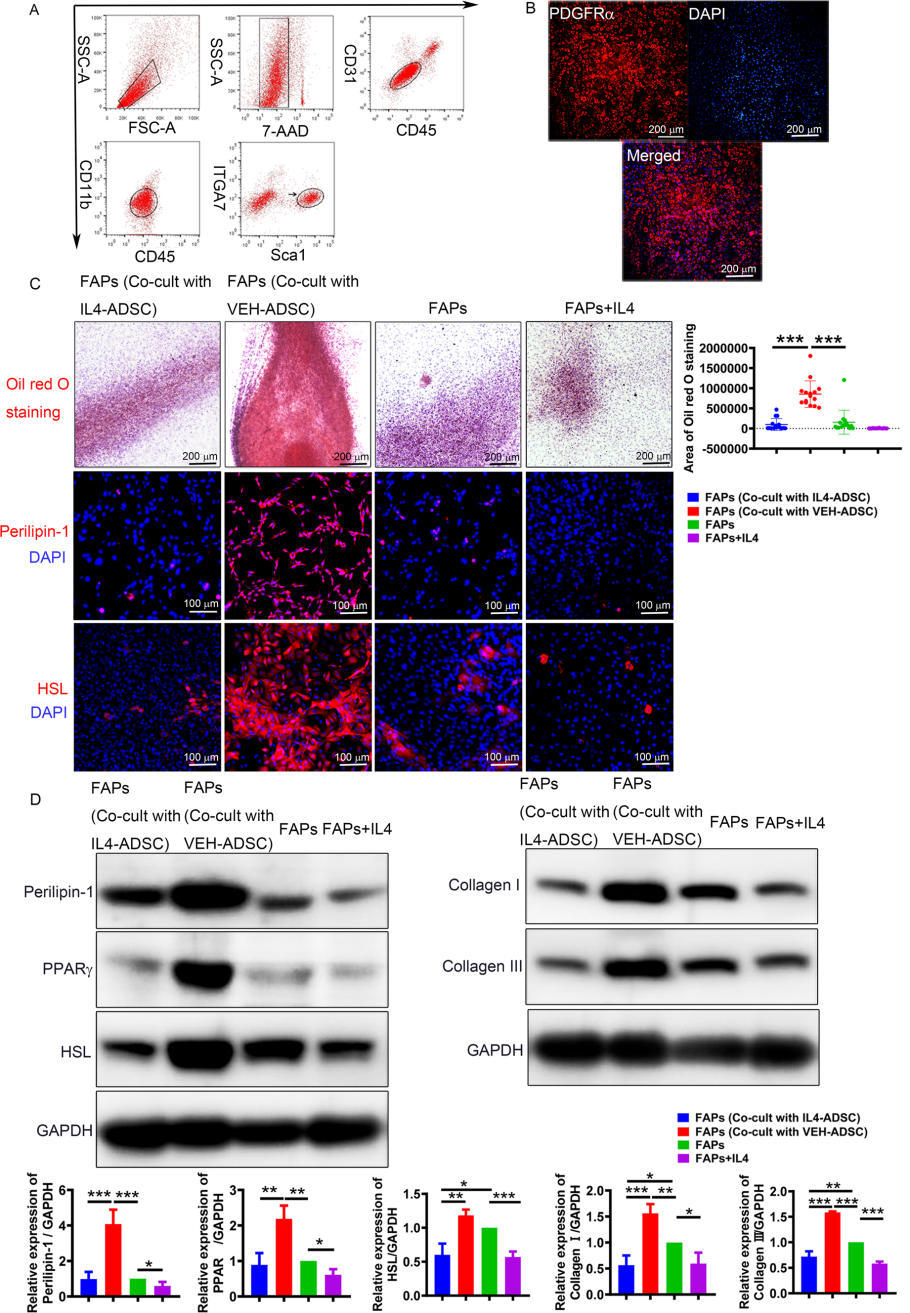
IL4-ADSC-derived myoblasts inhibit adipogenic and fibrogenic differentiation of FAPs, as indicated by the co-cultured experiment. **A** FACS enrichment scheme for FAPs of skeletal muscle of 8-week-old mdx mice. The black arrow indicates target cells. **B** Immuno-cyto-fluorescence analysis of PDGFRα (red) expression in FAPs. Nuclei were counterstained with DAPI (blue); scale bar, 200 μm. **C** Analysis of adipocytes forming in FAPs (Co-cult with IL4-ADSC), FAPs (Co-cult with VEH-ADSC), FAPs and FAPs + IL4 via Oil Red O staining; scale bar = 200 μm; and immune-cyto-fluorescence analysis of adipogenic markers, Perilipin-1 and HSL; scale bar = 100 μm. **D** Western blotting analysis of adipogenic markers, including Perilipin-1, PPARγ, HSL, and fibrogenic markers, including Collagen I and Collagen III; *P < 0.05, **P < 0.01, ***P < 0.001

FACS revealed that CD31^−^CD45^−^CD11b^−^ITGA7^−^Sca1^+^ was abundant in the limb muscles of mdx mice (Fig. 4A). Immuno-cyto-fluorescence indicated that 97.01 ± 0.89% of the sorted cells were positive for another specific marker, PDGFRα, consistent with previous findings [8] (Fig. 4B).

To investigate whether co-culturing with ADSC-derived-myoblasts and IL4 activation would affect the adipogenic and fibrogenic differentiation of FAPs, we analyzed the expression of fibrogenic and adipogenic genes in mono-cultured FAPs, IL4-treated FAPs (FAPs + IL4), and FAPs co-cultured with IL4-ADSC and VEH-ADSC-derived myoblasts [named FAPs (Co-cult with IL4-ADSC) and FAPs (Co-cult with VEH-ADSC), respectively]. Oil Red O staining revealed that FAPs (Co-cult with VEH-ADSC) form more lipid-laden adipocytes compared to mono-cultured FAPs and FAPs (Co-cult with IL4-ADSC) (Fig. 4C). Furthermore, there was no difference in the formation of adipocytes between mono-cultured FAPs and FAPs (Co-cult with IL4-ADSC), FAPs + IL4, respectively (Fig. 4C). We further analyzed the expression of adipogenic genes, including Perilipin-1, PPARγ, and HSL using immune-cyto-fluorescence (Fig. 4C) and western blotting (Fig. 4D). The expression levels of Perilipin-1, PPARγ, and HSL, which are markers of mature adipocytes, in FAPs (Co-cult with VEH-ADSC) were increased compared to those of FAPs (Co-cult with IL4-ADSC) and mono-cultured FAPs, but were decreased in FAPs + IL4 compared to mono-cultured FAPs, indicated by western blotting (Fig. 4D). The expression level of HSL in FAPs (Co-cult with IL4-ADSC) was significantly decreased compared to that in mono-cultured FAPs (Fig. 4D). The expression levels of Collagen I and Collagen III in FAPs (Co-cult with VEH-ADSC) were increased, compared to those in mono-cultured FAPs and FAPs (Co-cult with IL4-ADSC) (Fig. 4D). Furthermore, the expression levels of Collagen I and Collagen III in FAPs (Co-cult with IL4-ADSC) and FAPs + IL4 were decreased compared to those in mono-cultured FAPs (Fig. 4D). The above results illustrated that ADSC-derived myoblasts promote the adipogenic and fibrogenic differentiation of FAPs and that overexpression of IL4 in ADSC inhibits these effects.

### IL4-ADSC-derived myoblasts co-cultured with FAPs in vitro show better terminal myogenic differentiation ability

**Fig. 5:**
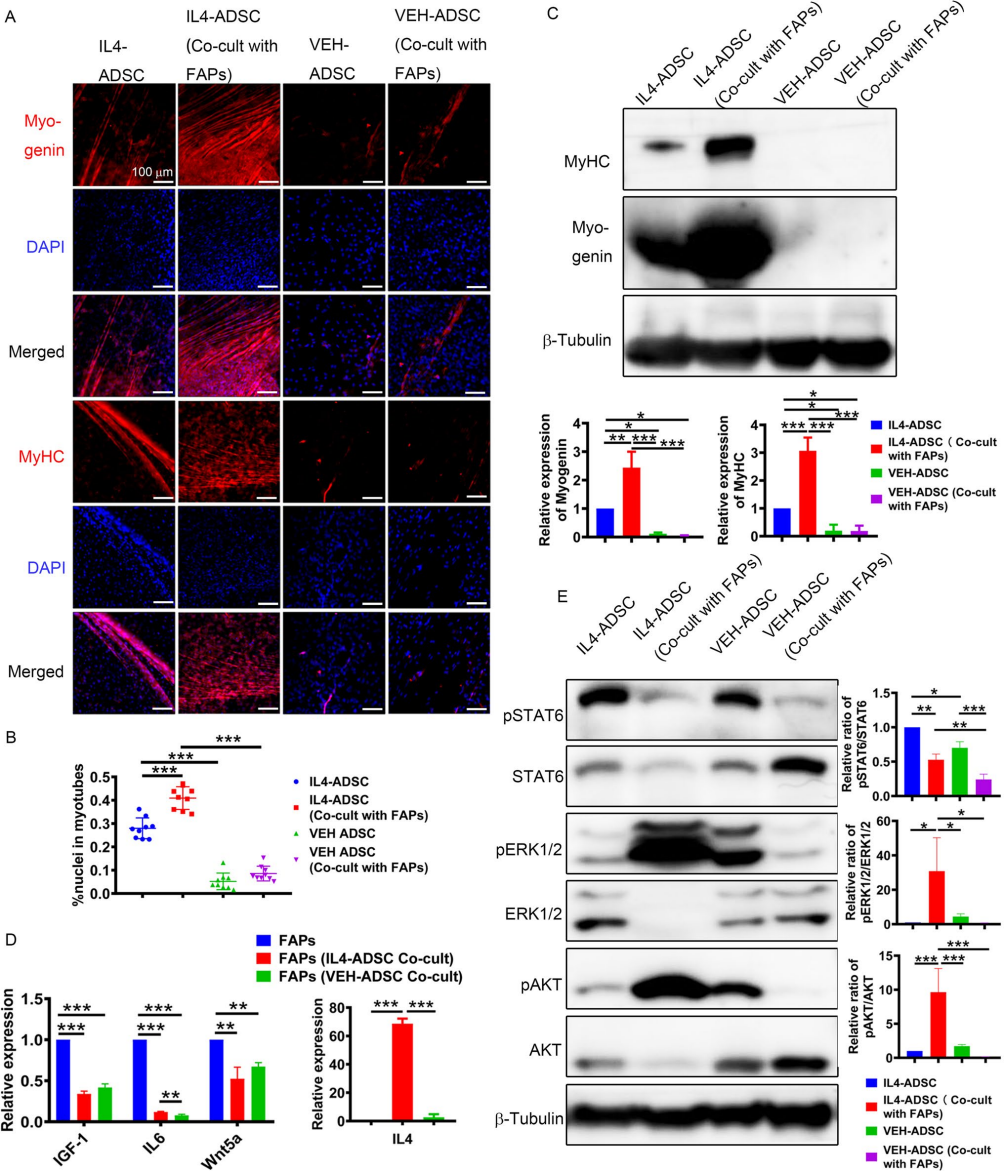
FAPs promote myogenic differentiation of IL4-ADSC-derived myoblasts but not of VEH-ADSC-derived myoblasts, as indicated by the co-cultured experiment. **A** Immuno-cyto-fluorescence analysis of Myogenin (red) and MyHC (red) expression in IL4-ADSC (Co-cult with FAPs), IL4-ADSC, VEH-ADSC (Co-cult with FAPs), and VEH-ADSC following myogenic differentiation for 28 d. Nucleus was counterstained with DAPI (blue); scale bar, 100 μm. **B** Fusion indexes of myotubes in IL4-ADSC (Co-cult with FAPs), IL4-ADSC, VEH-ADSC (Co-cult with FAPs) and VEH-ADSC were calculated following myogenic differentiation for 28 d. **C** Western blotting analysis of Myogenin and MyHC expression in IL4-ADSC (Co-cult with FAPs), IL4-ADSC, VEH-ADSC (Co-cult with FAPs) and VEH-ADSC following myogenic differentiation for 28 d. **D** qPCR analysis of IGF-1, IL-6, Wnt5a, and IL4 expression in FAPs (IL4-ADSC Co-cult) and FAPs (VEH-ADSC Co-cult) in co-cultured system after myogenic differentiation for 28 d and mono-cultured FAPs. **E** Western blotting analysis of phosphorylation ability of STAT6, AKT and ERK1/2; *P < 0.05, **P < 0.01, ***P < 0.001

To investigate whether FAPs affect the myogenesis of myoblasts derived from ADSC, we co-cultured FAPs with ADSC-derived myoblasts. The expression levels of Myogenin and MyHC in IL4-ADSC (Co-cult with FAPs) were dramatically increased compared to those in mono-cultured IL4-ADSC (Fig. 5A and C). No difference was observed between VEH-ADSC (Co-cult with FAPs) and mono-cultured VEH-ADSC (Fig. 5A and C). Similar results were observed when the fusion index was analyzed. Numerous myotube-like cells with MyHC-positive staining and multinucleated cells were observed after co-culturing, and the fusion index of the IL4-ADSC (Co-cult with FAPs) increased from 27.39% to 40.14%. In contrast, no difference was observed between the fusion indexes of VEH-ADSC (Co-cult with FAPs) and VEH-ADSC (Fig. 5B). The above results indicated that overexpression of IL4 enhances the ability of FAPs to promote the myogenesis of ADSC-derived myoblasts.

We speculated that FAPs may secrete cytokines that promote the myogenesis of IL4-ADSC-derived myoblasts. Therefore, we investigated the mRNA expression levels of several common myogenesis-promoting factors (IGF-1, IL-6, Wnt5a, Wnt3a, and Wnt1) and IL4 in co-cultured FAPs and mono-cultured FAPs (Fig. 5D). The expression levels of Wnt3a and Wnt1 in different FAPs groups were too low to be detected (data not shown). We found that although the expression of IL-6 in FAPs (IL4-ADSC Co-cult) was higher than that in FAPs (VEH-ADSC Co-cult), both were significantly lower than that in mono-cultured FAPs, indicating that IL-6 may not play a leading role in promoting the myogenesis of IL4-ADSC (Co-cult with FAPs). However, the IL4 mRNA level of FAPs (IL4-ADSC Co-cult) was significantly higher than that of FAPs (VEH-ADSC Co-cult), indicating that FAPs (IL4-ADSC Co-cult) in the co-cultured system secreted IL4 in large quantities, promoting myogenesis. To elucidate the pathways that may mediate the myogenic effects of IL4 in ADSC co-cultured with FAPs, we further investigated three major signaling pathways activated by IL4 in ADSC, namely STAT6, protein kinase AKT, and ERK1/2 (Fig. 5E). Phosphorylation of AKT and ERK1/2 in the IL4-ADSC (Co-cult with FAPs) was significantly increased compared to that in mono-cultured IL4-ADSC. By contrast, the phosphorylation of AKT and ERK1/2 in VEH-ADSC (Co-cult with FAPs) was decreased compared to those in mono-cultured VEH-ADSC, indicating that a dramatic change in the phosphorylation of AKT and ERK1/2 may have contributed to a dramatic improvement in the myogenesis of IL4-ADSC (Co-cult with FAPs), primarily because AKT and ERK pathways are known to be typically and essentially involved in improving myogenesis [27].

### Systemic delivery of IL4-ADSC-derived myoblasts restores better dystrophin expression in mdx mice

**Fig. 6:**
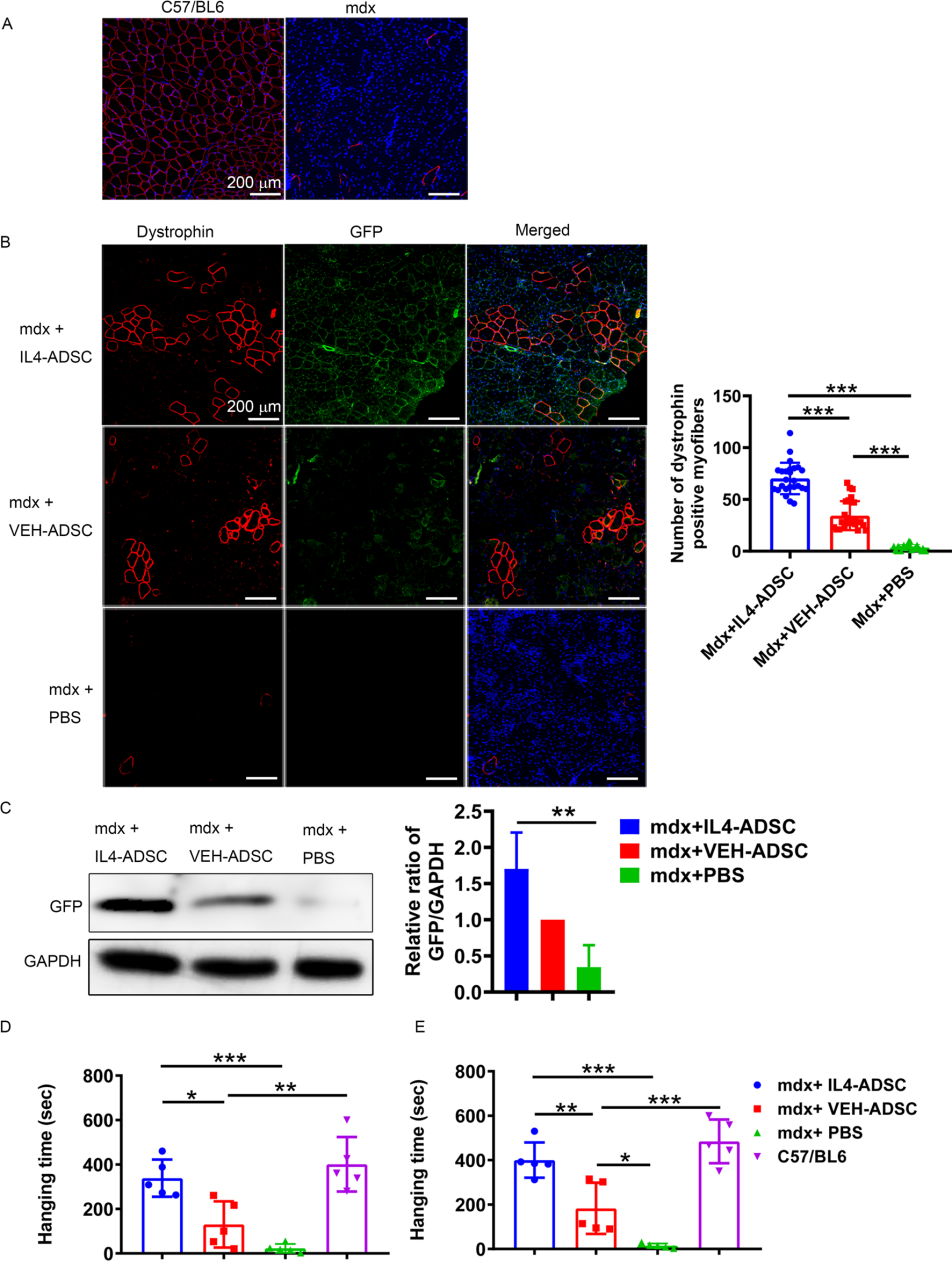
IL4-ADSC-derived myoblast transplantation further improves muscle dystrophin expression and motor ability in mdx mice. **A** Dystrophin expression in the TA of 24-week-old C57 and mdx mice. **B** Dystrophin expression in the TAs of mdx + IL4-ADSC, mdx + VEH-ADSC, and mdx + PBS mice at 16-week post-transplantation of ADSC. Red: dystrophin, green: GFP, blue: DAPI; scale bar, 200 μm. **C** GFP expression analyzed via western blotting in the TAs of mdx + IL4-ADSC, mdx + VEH-ADSC, and mdx + PBS mice at 16-week post-transplantation of ADSC (**D**, **E**) Hanging times for the fore-limb (**D**) and four-limb (**E**) hanging test were used to evaluate motor function in mice; *P < 0.05, **P < 0.01, ***P < 0.001

Dystrophin was evenly expressed in the myofibers of C57/BL6 mice. It was not expressed in the myofibers of mdx mice and only occasionally expressed in individual reverting fibers (Fig. 6A). The results of the current study showed that the dystrophin^+^ myofibers of mdx + IL4-ADSC and mdx + VEH-ADSC mice were significantly increased compared to those of mdx + PBS mice and appeared in pieces while dystrophin^+^ cells expressing GFP indicated that dystrophin^+^ muscle fibers had been newly formed by the transplanted donor cells (Fig. 6B). It was observed that the dystrophin^+^ myofibers in the TAs of mdx + IL4-ADSC mice were significantly increased compared to those in mdx + VEH-ADSC mice (Fig. 6B). Western blot of GFP in the TAs of different groups of mice also confirmed the recruitment of IL4-ADSC and VEH-ADSC to the TAs of mdx mice (Fig. 6C). The expression of GFP in the TAs of mdx + IL4-ADSC mice was higher than that in the TAs of mdx + VEH-ADSC mice (Fig. 6C).

### Systemic delivery of IL4-ADSC-derived myoblasts improves the motor ability of mdx mice

Sixteen weeks after transplantation, different groups of mdx mice were assessed for motor ability via hanging tests, with C57/BL6 mice as healthy controls. All mice survived until the end of the experiment. In both hanging tests involving forelimbs and four limbs, mdx + IL4-ADSC mice showed significantly better motor ability than mdx + VEH-ADSC and mdx + PBS mice (Fig. 6D, E). Mdx + VEH-ADSC mice exhibited better motor ability than mdx + PBS mice in the hanging tests with four limbs (Fig. 6 E).

### Systemic delivery of IL4-ADSC-derived myoblasts is more effective in preventing muscle fibrosis in mdx mice

**Fig. 7:**
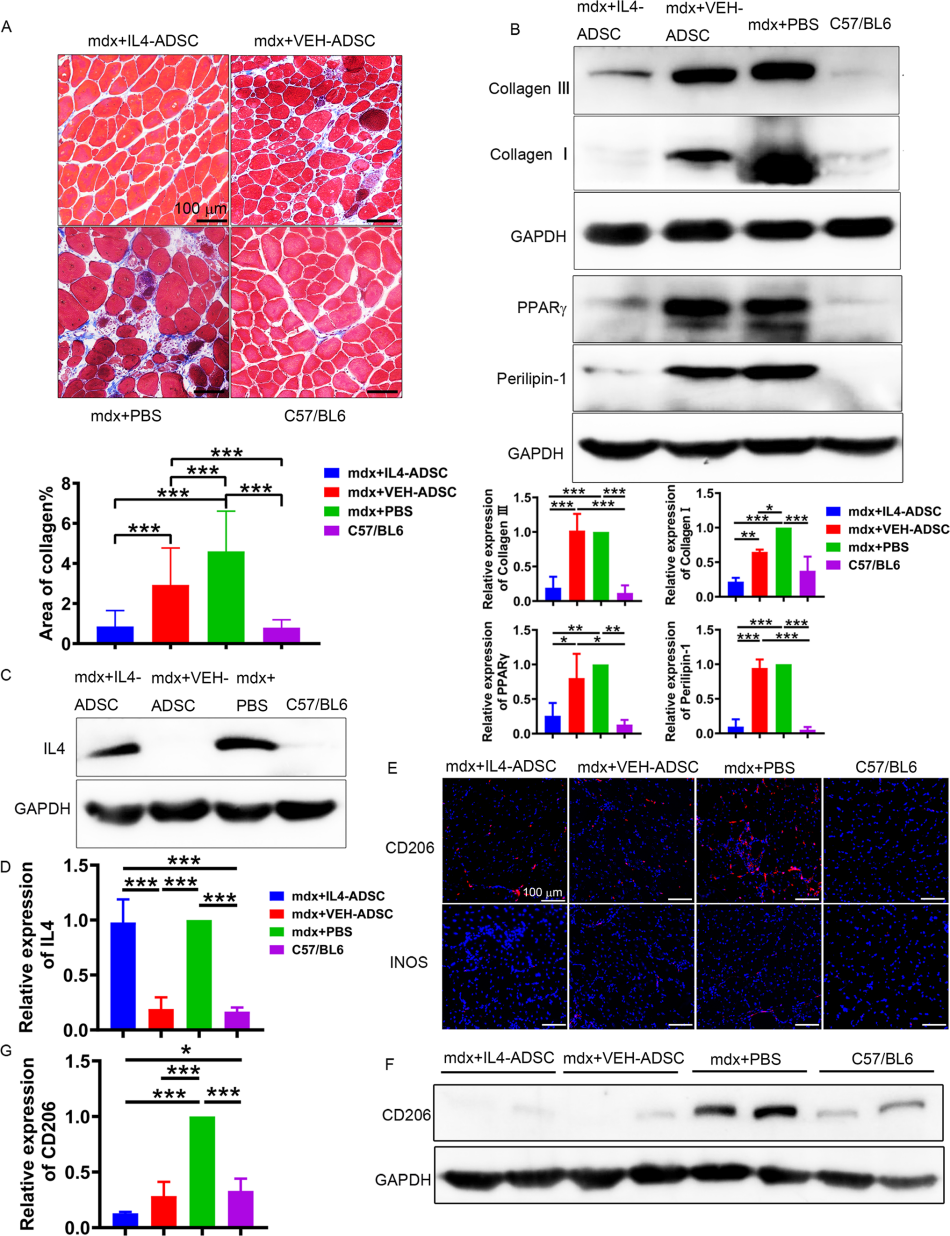
IL4-ADSC-derived myoblast transplantation further improves muscle fibrosis, adipocyte accumulation, and macrophage accumulation in mdx mice. **A** Muscle fibrosis was assessed using Masson's Trichrome Staining, which revealed collagen expression; scale bar, 100 μm. **B** Western blotting showing the expression of fibrogenic markers, including Collagen I and Collagen III, and adipogenic markers, including PPARγ and Perilipin-1, in the TAs of different groups of mice. **C**, **D** Western blotting showing the expression of IL4 in the TAs of different groups of mice. **E** Immuno-histo-fluorescence showing the expression of CD206 (red) and INOS (red) in TAs of different groups of mice. Nuclei were counterstained with DAPI (blue); scale bar, 100 μm. **F**, **G** Western blotting showing the expression of IL4 in TAs of different groups of mice; *P < 0.05, **P < 0.01, ***P < 0.001

Masson's trichrome staining revealed that fibrosis in the TAs of mdx + IL4-ADSC mice had decreased significantly compared to that of mdx + VEH-ADSC and mdx + PBS mice and that muscle fibrosis in mdx + IL4-ADSC had been reduced to the level of C57/BL6s (Fig. 7A). Muscle fibrosis in mdx + VEH-ADSC mice was also decreased compared to that of mdx + PBS mice (Fig. 7A). Similar observations were made via western blotting. The expression levels of Collagen I and Collagen III in the TAs of mdx + IL4-ADSC and mdx + VEH-ADSC mice were decreased compared to that in mdx + PBS mice, and their expression levels in mdx + IL4-ADSC had decreased more obviously than that of mdx + VEH-ADSC and decreased to reach the level of C57/BL6s (Fig. 7B). These results indicated that IL4-ADSC-derived myoblasts may be more effective in preventing muscle fibrosis in mdx mice than VEH-ADSC-derived myoblasts.

### Systemic delivery of IL4-ADSC-derived myoblasts is more effective in preventing muscle adipocyte infiltration in mdx mice

Besides fibrosis-related infiltration, adipocyte infiltration constitutes another prominent characteristic of DMD. To investigate whether the transplantation of IL4-ADSC and VEH-ADSC-derived myoblasts affects adipocyte infiltration in the muscles of mdx mice, we analyzed the expression levels of the mature adipocyte markers, Perilipin-1 and PPARγ, via western blotting. The expression levels of Perilipin-1 and PPARγ in the TA of the mdx + IL4-ADSC mice were significantly lower than those in mdx + VEH-ADSC and mdx + PBS mice, decreasing to reach the levels seen in C57/BL6s (Fig. 7B). The results indicated that IL4-ADSC-derived myoblasts were more effective in preventing adipocyte infiltration into the muscles of mdx mice than VEH-ADSC-derived myoblasts.

### Systemic delivery of IL4-ADSC-derived myoblasts is more effective in preventing muscle macrophage infiltration in mdx mice

IL4 levels in the TAs of mdx + PBS and mdx + IL4-ADSC mice were higher than those of C57/BL6s and mdx + VEH-ADSC (Fig. 7C, D). IL4 reportedly activates M2 macrophages [28]. Immunofluorescence indicated that macrophages in the TAs of mdx mice were mainly CD206-positive M2 macrophages. In contrast, INOS-positive M1 macrophages were scarce in each group, a finding consistent with that of a previous study [29] (Fig. 7E). Western blotting results showed that although CD206 expression levels in the TAs of mdx + IL4-ADSC and mdx + VEH-ADSC mice did not exhibit statistical differences, they were still significantly lower than those in mdx + PBSs and similar to the expression levels of C57/BL6s (Fig. 7F, G). INOS expression in the TAs of different groups was too low to be detected via western blotting (Data not shown). The above data showed that transplantation of IL4-ADSC and VEH-ADSC-derived myoblasts had significantly improved macrophage infiltration in dystrophic muscles and that an increase in the level of IL4 in mdx + IL4-ADSC muscles had not increased the distribution of M2 macrophages.

## Discussion

In the present study, we have provided evidence indicating the potential of IL4-ADSC as a therapeutic approach for DMD treatment. Our results showed that IL4 overexpression had significantly improved the myogenic capacity of ADSC in vitro. Furthermore, we detected crosstalk between ADSC-derived-myoblasts and muscle-resident FAPs via in vitro co-culture. Overexpression of IL4 altered the relationship between ADSC-derived myoblasts and FAPs, causing FAPs to promote myogenesis in ADSC, while inhibiting differentiation of FAPs. The in vivo study confirmed the potential of IL4-ADSC treatment to improve post-transplantation therapeutic effects in mdx mice, via restoring dystrophin-positive myofibers and preventing muscle fibrosis and intramuscular adipocytes.

Any limitation of myogenesis potential lowers the efficacy of ADSC therapy in dystrophic mice. Terminal myogenesis of ADSC in vitro evidently relies on co-culture with primary myoblasts [30–32]. Promoting terminal myogenesis may improve the therapeutic effect of ADSC therapy in DMD. The exact mechanism underlying the myogenesis program of ADSC has yet to be determined. Our results indicate that IL4 overexpression significantly improves terminal myogenesis of ADSC in vitro via a pathway that is not involved in the expression of MYFs during the early stages. The role played by the IL4 pathway in myogenesis is not yet clear but mainly focuses on improving myoblast fusion and the migration of myogenic precursor cells [17–19]. Although IL4 treatment reportedly improved the ability of ADSC to proliferate and fuse with myoblasts when co-cultured with C2C12 myoblasts, it did not affect the expression of myogenic markers, such as Myod1 and Myf5, in ADSC [21, 22]. Moreover, IL4 overexpression did not affect the expression levels of Myf5 and Myod1; however, it significantly improved terminal myogenesis and the expression of Pax7, a marker of muscle progenitors in ADSC, via activation of the IL4 pathway. These differences may be attributed to myogenic differentiation being induced for an extended period and the expression of related myogenic markers being tested at different time points.

FAPs play an essential role in muscle regeneration through their interaction with muscle SCs, which inhibit the adipogenesis of FAPs during normal repairs that follow acute injuries [8, 11]. DMD-related chronic muscle damage alters the interactions between FAPs and SCs. Myotube-dependent inhibition of FAPs differentiation is altered in DMD patients due to myogenic progenitors (MPs) inhibiting adipogenesis and promoting the fibrogenesis of FAPs in co-culture [33]. The IL4 pathway is the switch that determines the function of FAPs in acute muscle injury [15]. The role of the IL4 pathway in FAPs of dystrophic muscles is not yet clear. Studies have mainly focused on the role of HDAC inhibitor (HDACi)-mediated signaling pathways in regulating FAPs function in dystrophic muscle. FAPs mediate the ability of HDACi to promote dystrophic muscle regeneration by facilitating myogenesis in the SCs of young mdx mice, but not in old mdx mice [34]. Further studies have indicated that HDACi may regulate the fate of FAPs in the muscles of young mdx mice by regulating the expression of myogenic-related micro-RNAs in FAPs [10, 35]. A recent study has indicated that type II innate immunity may influence the balance between regenerative and fibrotic responses during muscular dystrophy via the FAPs/ILC2/eosinophil axis [36]. This finding is in accordance with our study, because we have also found that the IL4 pathway, a component of type II innate immunity, regulates the balance between myoblast regeneration and fibrotic responses.

It appears that studies that describe possible relationships between engrafted stem cells and FAPs in dystrophic muscle are yet to be conducted. While being consistent with those of previous studies to some extent, our findings have led to some novel discoveries. We detected crosstalk between ADSC-derived-myoblasts and FAPs, and confirmed myotube-dependent promotion of FAPs differentiation via co-culture. Our study differs in that the myoblasts were derived from ADSC that are actively used in cell therapy, indicating the importance of focusing on the crosstalk between engrafted cells and FAPs in skeletal muscle. Overall, the complicated interaction between myoblasts originating from ADSC and FAPs may affect the transplantation effect of ADSC-derived myoblasts in vivo. Activation of IL4 in FAPs stimulates their ability to promote the myogenesis of SCs and inhibits their differentiation into adipocytes via STAT6 signaling in muscle [15]. The current study confirmed that overexpression of IL4 dramatically altered the two-way interaction between FAPs and myoblasts derived from ADSC.

Improvement in motor function is an essential indicator of the effect of DMD treatment. The apparent improvements caused by the transplanted IL4-ADSC-derived myoblasts may be beneficial from multiple aspects as follows: (i) significantly improved terminal myogenesis caused by IL4-ADSC-derived myoblasts; (ii) the obviously enhanced effect of IL4-ADSC-derived myoblast transplantation on muscle fibrosis via activation of the IL4 pathway in FAPs, compared to that of VEH-ADSC-derived myoblasts; (iii) significantly less infiltration of muscle by mdx + IL4-ADSC compared to that of mdx + VEH-ADSC, due to IL4 activation inhibiting the adipogenic differentiation of FAPs promoted by ADSC-derived-myoblasts. For the first time, we report that overexpression of IL4 effectively improves the effect of ADSC therapy on muscle fat infiltration in mdx mice, thereby providing a new concept in ADSC therapy that may help enhance muscle fat infiltration in mdx mice.

We found that IL4 expression in the TA of 6-month-old mdx mice was increased compared to that of C57/BL6 mice, which was consistent with previous reports indicating that the regenerative stage of mdx mouse limb muscles was possibly associated with infiltration by T cells or eosinophils [29, 37]. Elevated IL4 directly boosts the regenerative capacity of muscle and satellite cells on the one hand [19] and polarizes macrophages to the M2 type on the other hand [38]. M2 macrophages are reportedly associated with muscle fibrosis in mdx mice [39]. In conclusion, the elevated expression of IL4 in mdx muscle exerts a double-edged effect. Interestingly, transplantation of IL4-ADSC not only elevated the production of IL4 but also reduced infiltration by macrophages, thereby attenuating the harmful effects exerted by M2 macrophage (iNOS- CD206 +) infiltration. The decreased infiltration of muscle macrophages possibly contributed to the treatment effect of ADSC therapy, as indicated by low CD206 expression in both mdx + IL4-ADSC and mdx + VEH-ADSC. Together, these results indicated that IL4-ADSC therapy may play a positive role in dystrophic muscle therapy.

This study was affected by certain limitations. Firstly, we simply focused on the relationship between engrafted cells and FAPs, and failed to investigate the benefits conferred by the in vivo study to other mechanisms. Further research should focus on elucidating more complicated interactions that may exist between other cell types. Furthermore, the improved post-transplantation therapeutic effects exerted by IL4-ADSC on mdx mice cannot be said to have benefited from the altered crosstalk between engrafted IL4-ADSC and FAPs or from the improved myogenic impact of IL4-ADSC themselves, although the in vitro co-culture study of IL4-ADSC and FAPs does offer some evidence. Besides, many studies show the detrimental effects of promoting the activity and generation of this cytokine, as it is an immunosuppressor of several cell activities. In this regard, IL4 has been shown to worsen the outcome of important diseases, such as cancer, virus, and parasitic diseases [40, 41]. Thus, this particular therapeutic alternative for muscular dystrophy cannot be used in some clinical settings.

## Conclusion

To the best of our knowledge, this is the first study to determine the extra benefits conferred by a combination therapy involving IL4 administration and cell therapy on the positive effects exerted by muscle micro-environment cells on the regenerative therapy of DMD. In conclusion, IL4 overexpression improves the therapeutic potential of ADSC transplantation in mdx mice, possibly by altering crosstalk between engrafted stem cells and resident FAPs and improving the myogenesis efficacy of IL4-ADSC.

## Data Availability

The datasets supporting our findings are presented in the article.

## Abbreviations

DMD: Duchenne muscular dystrophy
FAPs: Fibro-/adipogenic progenitors
PDGFR-α: Platelet-derived growth factor receptor-α
Sca1: Stem cell antigen 1
SCs: Satellite cells
ADSC: Adipose-derived stem cells
FACS: Fluorescence-activated cell sorting
bFGF: Basic fibroblast growth factor-basic
GFP: Green fluorescent protein
MOI: Multiplicity of infection
ELISA: Enzyme-linked immunosorbent assay
CCK8: Cell counting kit 8
qPCR: Quantitative polymerase chain reaction
MPs: Myogenic progenitors
HDACi: HDAC inhibitor

## References

[1] Klingler W, Jurkat-Rott K, Lehmann-Horn F, Schleip R (2021). The role of fibrosis in Duchenne muscular dystrophy. Acta Myol.

[2] Dubuisson N, Versele R, Planchon C (2022). Histological methods to assess skeletal muscle degeneration and regeneration in Duchenne muscular dystrophy. Int J Mol Sci.

[3] Foidart M, Foidart JM, Engel WK (1981). Collagen localization in normal and fibrotic human skeletal muscle. Arch Neurol.

[4] Biressi S, Miyabara EH, Gopinath SD (2014). A Wnt-TGFbeta2 axis induces a fibrogenic program in muscle stem cells from dystrophic mice. Sci Transl Med.

[5] Pessina P, Kharraz Y, Jardí M (2015). Fibrogenic cell plasticity blunts tissue regeneration and aggravates muscular dystrophy. Stem Cell Rep.

[6] Wang XY, Chen JM, Homma ST (2022). Diverse effector and regulatory functions of fibro/adipogenic progenitors during skeletal muscle fibrosis in muscular dystrophy. iScience.

[7] Giuliani G, Rosina M, Reggio A (2022). Signaling pathways regulating the fate of fibro/adipogenic progenitors (FAPs) in skeletal muscle regeneration and disease. FEBS J.

[8] Joe AWB, Yi L, Natarajan A (2010). Muscle injury activates resident fibro/adipogenic progenitors that facilitate myogenesis. Nat Cell Biol.

[9] Lemos DR, Babaeijandaghi F, Low M (2015). Nilotinib reduces muscle fibrosis in chronic muscle injury by promoting TNF-mediated apoptosis of fibro/adipogenic progenitors. Nat Med.

[10] Sandona M, Consalvi S, Tucciarone L (2020). HDAC inhibitors tune miRNAs in extracellular vesicles of dystrophic muscle-resident mesenchymal cells. EMBO Rep.

[11] Uezumi A, Fukad S, Yamamoto N (2010). Mesenchymal progenitors distinct from satellite cells contribute to ectopic fat cell formation in skeletal muscle. Nat Cell Biol.

[12] Moratal C, Raffort J, Arrighi N (2018). IL-1beta- and IL-4-polarized macrophages have opposite effects on adipogenesis of intramuscular fibro-adipogenic progenitors in humans. Sci Rep.

[13] Reggio A, Rosina M, Palma A (2020). Adipogenesis of skeletal muscle fibro/adipogenic progenitors is affected by the WNT5a/GSK3/beta-catenin axis. Cell Death Differ.

[14] Wosczyna MN, Konishi CT, Perez CEE (2019). Mesenchymal stromal cells are required for regeneration and homeostatic maintenance of skeletal muscle. Cell Rep.

[15] Heredia JE, Mukundan L, Chen FM (2013). Type 2 innate signals stimulate fibro/adipogenic progenitors to facilitate muscle regeneration. Cell.

[16] Dong Y, Silva KA, Dong Y, Zhang L (2014). Glucocorticoids increase adipocytes in muscle by affecting IL-4 regulated FAPs activity. FASEB J.

[17] Horsley V, Jansen KM, Mills ST, Pavlath GK (2003). IL-4 acts as a myoblast recruitment factor during mammalian muscle growth. Cell.

[18] Lafreniere JF, Mills P, Bouchentouf M, Tremblay JP (2006). Interleukin-4 improves the migration of human myogenic precursor cells in vitro and in vivo. Exp Cell Res.

[19] Costamagna D, Duelen R, Penna F (2020). Interleukin-4 administration improves muscle function, adult myogenesis, and lifespan of colon carcinoma-bearing mice. J Cachexia Sarcopenia Muscle.

[20] Zhang Y, Zhu Y, Li Y (2015). Long-term engraftment of myogenic progenitors from adipose-derived stem cells and muscle regeneration in dystrophic mice. Hum Mol Genet.

[21] Cai Y, Li JY, Jia CS (2020). Therapeutic applications of adipose cell-free derivatives: a review. Stem Cell Res Ther.

[22] Archacka K, Bem J, Brzoska E (2020). Beneficial effect of IL-4 and SDF-1 on myogenic potential of mouse and human adipose tissue-derived stromal cells. Cells.

[23] Zimowska M, Archacka K, Brzoska E (2020). IL-4 and SDF-1 increase adipose tissue-derived stromal cell ability to improve rat skeletal muscle regeneration. Int J Mol Sci.

[24] Farup J, Madaro L, Puri PL, Mikkelsen UR (2015). Interactions between muscle stem cells, mesenchymal-derived cells and immune cells in muscle homeostasis, regeneration and disease. Cell Death Dis.

[25] Wang L, Li H, Lin J (2021). CCR2 improves homing and engraftment of adipose-derived stem cells in dystrophic mice. Stem Cell Res Ther.

[26] Aartsma-Rus A, van Maaike PM (2014). Assessing functional performance in the mdx mouse model. J Vis Exp.

[27] Wang J, Tan J, Qi Q (2018). miR-487b-3p Suppresses the proliferation and differentiation of myoblasts by targeting IRS1 in skeletal muscle myogenesis. Int J Biol Sci.

[28] Varin A, Mukhopadhyay S, Herbein G, Gordon S (2010). Alternative activation of macrophages by IL-4 impairs phagocytosis of pathogens but potentiates microbial-induced signalling and cytokine secretion. Blood.

[29] Villalta SA, Nguyen HX, Deng B (2009). Shifts in macrophage phenotypes and macrophage competition for arginine metabolism affect the severity of muscle pathology in muscular dystrophy. Hum Mol Genet.

[30] Di-Rocco G, Iachininoto MG, Tritarelli A (2006). Myogenic potential of adipose-tissue-derived cells. J Cell Sci.

[31] Eom YW, Lee JE, Yang MS (2011). Effective myotube formation in human adipose tissue-derived stem cells expressing dystrophin and myosin heavy chain by cellular fusion with mouse C2C12 myoblasts. Biochem Biophys Res Commun.

[32] Vieira NM, Brandalise V, Zucconi E (2008). Human multipotent adipose-derived stem cells restore dystrophin expression of Duchenne skeletal-muscle cells in vitro. Biol Cell.

[33] Moratal C, Arrighi N, Dechesne CA, Dani C (2019). Control of muscle fibro-adipogenic progenitors by myogenic lineage is altered in aging and Duchenne muscular dystrophy. Cell Physiol Biochem.

[34] Mozzetta C, Consalvi S, Saccone V (2013). Fibroadipogenic progenitors mediate the ability of HDAC inhibitors to promote regeneration in dystrophic muscles of young, but not old Mdx mice. EMBO Mol Med.

[35] Saccone V, Consalvi S, Giordani L (2014). HDAC-regulated myomiRs control BAF60 variant exchange and direct the functional phenotype of fibro-adipogenic progenitors in dystrophic muscles. Genes Dev.

[36] Kastenschmidt JM, Coulis G, Farahat PK (2021). A stromal progenitor and ILC2 niche promotes muscle eosinophilia and fibrosis-associated gene expression. Cell Rep.

[37] Sek AC, Moore IN, Smelkinson MG (2019). Eosinophils do not drive acute muscle pathology in the mdx mouse model of Duchenne muscular dystrophy. J Immunol.

[38] Basil JP (2022). Macrophage plasticity in Duchenne muscular dystrophy: a nexus of pathological remodelling with therapeutic implications. J Physiol.

[39] Vidal B, Serrano AL, Tjwa M (2008). Fibrinogen drives dystrophic muscle fibrosis via a TGFβ/alternative macrophage activation pathway. Genes Dev.

[40] Kwaśniak K, Czarnik-Kwaśniak J, Maziarz A (2019). Scientific reports concerning the impact of interleukin 4, interleukin 10 and transforming growth factor β on cancer cells. Cent Eur J Immunol.

[41] Maksoud S, El Hokayem J (2023). The cytokine/chemokine response in Leishmania/HIV infection and co-infection. Heliyon.

